# Vitamin D level, lipid profile, and vitamin D receptor and transporter gene variants in sickle cell disease patients from Kurdistan of Iraq

**DOI:** 10.1002/jcla.23908

**Published:** 2021-07-14

**Authors:** Abdalla Hussein Hama, Ebrahim Shakiba, Zohreh Rahimi, Mehran Karimi, Hadi Mozafari, Omed Adnan Abdulkarim

**Affiliations:** ^1^ Department of Clinical Biochemistry Medical School Kermanshah University of Medical Sciences Kermanshah Iran; ^2^ Behavioral Disease Research Center Medical School Kermanshah University of Medical Sciences Kermanshah Iran; ^3^ Hematology Research Center Shiraz University of Medical Sciences Shiraz Iran; ^4^ Jin Pediatric Hematology Oncology Center Duhok Iraq

**Keywords:** GC, lipid profile, SCD, VDR FokI, VDR TaqI, vitamin D

## Abstract

**Introduction:**

Sickle cell disease (SCD) patients are susceptible to the development of vitamin D deficiency (VDD). Vitamin D through binding to vitamin D receptor (VDR) exerts its function and affects gene transcription in target tissues. VDR gene variants affect bone mineral density.

**Methods:**

In a case‐control study, 101 SCD patients including 61 sickle cell anemia (SCA), 39 S/β‐thalassemia, and 1 HbS/HbD (SD) along with 110 healthy individuals from Kurdistan of Iraq were studied. The lipid profile, vitamin D level, FokI, and TaqI variants of VDR and group‐specific component (GC) were detected using the standard enzymatic method, the immunodiagnostic systems limited EIA kit and PCR‐RFLP methods, respectively.

**Results:**

Around 93% and 82% of SCA and S/β‐thalassemia patients, respectively, had VDD compared to 83% of healthy individuals. Severe VDD (<10 ng/ml) was detected in 78.7% of patients with HbSS. Plasma levels of total cholesterol, HDL‐C, and LDL‐C in SCD patients were significantly lower compared to controls. Vitamin D levels were negatively correlated to TG and positively correlated to total cholesterol and HDL‐C. The frequencies of the C allele of FokI were 81.7% (*p* = 0.003), 80.3% (*p* = 0.034), and 84.6% (*p* = 0.011) in all SCD, SCA, and S/β‐thalassemia patients, respectively, compared to 69.1% in controls. However, no significant difference was detected comparing the frequencies of VDR TaqI and GC polymorphisms between SCD patients and controls.

**Conclusion:**

In the present study, we found hypocholesterolemia, high prevalence of VDR FokI C allele, and low vitamin D levels among children and adults with SCD from Kurdistan of Iraq.

## Graphical abstract

We found positive association between vitamin D level with HDL‐C and total cholesterol in studied individuals, induced hypocholesterolemia by hemolytic stress, the presence of severe vitamin D deficiency, and the high prevalence of VDR FokI C allele with an adverse effect on bone mineral density among SCD patients from Kurdistan of Iraq.

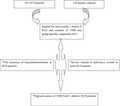

## 1 INTRODUCTION

The mutation of β^S^ (Codon 6 Glu > Val) in a homozygous state causes a serious illness, sickle cell anemia (SCA), with a generally shortened life span. The severity of the combined heterozygote state of Hb S/β‐thalassemia is variable according to the amount of Hb A production.^1^

In SCA and homozygous β‐thalassemia patients compared to normal individuals decreased cholesterol concentrations have been reported. The hemolytic stress was associated with a significant reduction in plasma lipid levels [total cholesterol, low‐density lipoprotein‐cholesterol (LDL‐C) and high‐density lipoprotein‐cholesterol (HDL‐C)] except for triglycerides (TG) in SCA and sickle/β‐thalassemia patients compared to sickle cell trait and normal individuals.^1^ Also, among children and adolescents with major β‐thalassemia, the levels of total cholesterol, LDL‐C, and HDL‐C were significantly lower and the TG level was significantly higher compared to healthy controls.^2^

Vitamin D deficiency (VDD) is a major global health problem. In SCA patients, VDD is highly prevalent, reaching up to 96% of the population.^3^ SCA patients are susceptible to the development of VDD due to decreased appetite or reduced nutrient absorption, increased basal metabolic rate with higher nutritional demands to compensate anemia, decreased conversion of vitamin D to its active form due to renal impairment, and lower levels of vitamin D binding protein levels in the inflammatory state of SCA. So, it is important to assess the vitamin D levels in SCA patients especially in children.^4^ VDD may contribute to too many complications of SCA. Muscle and bone pain may mimic acute sickle cell pain or chronic pain syndrome. Also, some bone complications of SCA may be caused or at least exacerbated by VDD.^3^ Further, in SCA, respiratory infection and asthma may lead to respiratory complications that are the leading cause of morbidity and mortality. Vitamin D has various immune functions including immune tolerance and the control of the adaptive and innate immune responses as in macrophages, monocytes, and B and T lymphocytes the expression of VDR increases considerably in response to inflammatory and immunological stimuli.^5^, ^6^ Vitamin D deficiency is associated with susceptibility to severe infections, and in children association between hypovitaminosis D and respiratory infection especially tuberculosis has been reported.^5^

The levels of 25(OH)‐D are influenced by genetic variation in receptors and other components involved in vitamin D metabolism.^7^

The carrier of vitamin D and its metabolites in the circulation is vitamin D binding protein that is encoded by a gene named group‐specific component (GC). The active form of vitamin D, 1, 25 (OH) 2‐D3 binds to the nuclear vitamin D receptor (VDR) that performs a heterodimer with the retinoid‐X receptor (RXR) and exerts the vitamin D function through influence gene transcription in target tissues.^8^ Most of the roles of vitamin D3 are through VDR‐RXR that activates VDR response elements in the promoter region of about 900 genes. Some non‐genomic rapid actions of vitamin D3 are mediated by the surface receptors.^9^

The present study aimed to detect the vitamin D level, lipid profile, and VDR variants of FokI, TaqI, and also GC variants in sickle cell disease (SCD) patients from Kurdistan of Iraq.

## 2 MATERIALS AND METHODS

### 2.1 Sample

We studied 101 SCD patients including 57 males and 44 females [61 with HbSS (15.9 ± 9.6 years), 39 S/β‐thalassemia (15.8 ± 7.9 years), and a 6‐year‐old child compound heterozygote for both HbS and HbD (HbSD)] along with 110 healthy individuals (59 males and 51 females) with the mean age of 15.3 ± 8.5 years from Duhok‐Jincenter for pediatric hemato‐oncology and Qaladze Public Hospital in Kurdistan of Iraq. Informed written consent was obtained from patients or the next‐of‐kin. The Ethics Committee of Kermanshah University of Medical Sciences approved the study and the study was in accordance with the principles of the Declaration of Helsinki II.

Among patients 62 individuals (61.4%) aged ≤18 years and in controls 68 (61.8%) subjects aged ≤18 years. Diagnosis of patients was based on laboratory tests and molecular analysis using the Dde I restriction enzyme for detection of the presence of the sickle gene.

### 2.2 Biochemical analysis

#### 2.2.1 Lipids

Total serum cholesterol (TC), TG, and HDL‐C levels were measured by the standard enzymatic method (Pars Azmoon kit, Tehran, Iran). The serum LDL‐C was calculated as follows: LDL‐C = total cholesterol − (HDL‐C + TG/5).

#### 2.2.2 Vitamin D

Vitamin D status was detected by the measurement of the serum level of 25 (OH) ‐D, the circulating form of the vitamin D, using the immunodiagnostic systems limited EIA kit. Severe vitamin D deficiency was defined as the level ≤10 ng/ml, the deficiency was defined as the level >10 to ≤20 ng/ml, and insufficiency was defined as level >20 to <30 ng/ml.^10^

### 2.3 Genotyping

The phenol‐chloroform method was used for the extraction of DNA from the leukocyte of the EDTA‐treated whole blood.^11^

The FokI T>C (rs2228570) in exon 2 and the TaqI T>C (rs731236) in exon 9 of the VDR gene were detected by polymerase chain reaction‐restriction fragment length polymorphism (PCR‐RFLP) method as previously described.^12^ The GC rs7041 was identified by the PCR‐RFLP method using the HaeIII restriction enzyme.^8^

### 2.4 Statistical analysis

The significance of differences in the frequencies of genotypes and alleles of FokI, TaqI, and GC polymorphisms between groups was calculated using the Chi‐square test. The Odds ratios (OR) as the estimates of relative risk for the disease were calculated, and 95% confidence intervals (CI) were detected by SPSS logistic regression software. A two‐tailed Student *t* test was used for comparing quantitative variables. The SPSS (SPSS Inc., Chicago, IL) statistical software package version 16.0 was used for statistical analysis. Statistical significance was considered at *p *< 0.05.

## 3 RESULTS

### 3.1 Hematological characteristics

Table 1 indicates the hematological characteristics of SCD patients. The HbS levels were 66 ± 14.9, 63 ± 14.6, and 28.8%, in SS, S/β‐thalassemia, and SD patients, respectively. The HbF levels were 19.8 ± 17.3, 19.1 ± 12.1, and 24.8%, in SS, S/β‐thalassemia, and SD patients, respectively.

**TABLE 1:** Hematological characteristics of studied patients

Patients (*n*)	Age	Hb (g/L)	%HCT	RBC (10^12^/L)	%HbA2	%HbF	%HbS	MCV (fl)	MCH (pg)
All (101)	15.83 ± 8.92	86.2 ± 13.4	25.2 ± 4.5	3.05 ± 0.84	3.38 ± 1.3	19.6 ± 15.3	64.4 ± 15.2	85.6 ± 15.8	29.6 ± 6.0
SS (61)	15.90 ± 9.6	83.8 ± 11.1	24.0 ± 3.8	2.6 ± 0.5	2.7 ± 0.9	19.8 ± 17.3	66.0 ± 14.9	92.0 ± 13.4	32.6 ± 4.9
S/β‐thalassemia (39)	15.8 ± 7.9	89.5 ± 15.5	26.9 ± 4.9	3.7 ± 0.8	4.5 ± 1.4	19.1 ± 12.1	63.0 ± 14.6	74.3 ± 12.6	24.8 ± 4.4
SD (1)	6.00	65	19.3	2.18	1.3	24.8	28.8	88.5	30.2
Controls (110)	15.3 ± 8.5	135.5 ± 14.5	39.7 ± 4.2	4.9 ± 0.5	‐	‐	‐	82.2 ± 4.7	28.2 ± 2.1

Parameters are described as Mean ± SD

### 3.2 Vitamin D level and lipid profile

The level of vitamin D was significantly lower (10.8 ± 5.7 ng/ml, *p *= 0.007) comparing all SCD patient's to controls (13.5 ± 8.4 ng/ml). The vitamin D levels were 9.7 ± 5, 12.5 ± 6.5, and 7.9 in patients with HbSS, S/β‐thalassemia, and HbSD patients, respectively (Table 2). The level of vitamin D was significantly lower in patients with HbSS than in controls (*p *< 0.001). Fifty‐seven out of 61 HbSS patients (93.4%) had vitamin D levels ≤20 ng/ml. In S/β‐thalassemia patients 32 out of 39 patients (82.1%) had vitamin D levels ≤20 ng/ml. In controls, 91 out of 110 individuals (82.7%) had vitamin D deficiency (≤20 ng/ml). Severe vitamin D deficiency (<10 ng/ml) was detected in 70 out of 101 SCD patients (69.3%) compared to 52 out of 110 controls (47.3%, *p* = 0.001). In SCA patients, 48 out of 61 patients (78.7%) had severe vitamin D deficiency.

**TABLE 2:** Vitamin D level and lipid profile in patients and controls

Group	*N*	Age (Years)	Vitamin D (ng/ml)	Total cholesterol (mg/dl)	Triglyceride (mg/dl)		HDL‐C (mg/dl)	LDL‐C (mg/dl)
HbSS	61	15.9 ± 9.6	9.7 ± 5*	103.1±20.5*	96.2 ± 27.9		34.0 ± 9.1*	49.2 ± 20.7*
Male	35	13.6 ± 7.9	10.2 ± 4.7**	102.5 ± 18.3*	89.7 ± 28.6		33.9 ± 10.4*	49.7 ± 17.2**
Female	26	19.0 ± 10.8	8.9 ± 5.3	104.6 ± 23.3*	105.1 ± 24.8		34.2 ± 7.3*	48.6 ± 24.9**
S/thal	39	15.8 ± 7.9	12.5 ± 6.5	104.0 ± 19.8*	93.2 ± 36.6		34.5 ± 10.1*	51.0 ± 17.3**
Male	21	14.5 ± 9.1	12.7 ± 6.7	102.6 ± 19.80*	92.43 ± 38.8		35.0 ± 10.1*	48.8 ± 17.0**
Female	18	17.3 ± 6.2	12.2 ± 6.3	106.2 ± 20.20*	94.2 ± 35.0		33.8 ± 10.4*	53.6 ± 17.9
HbSD	1							
Male	1	6.0	7.9	68**	126		30	12.8**
Female	‐	‐	‐	‐	‐		‐	‐
Controls	110	15.3 ± 8.5	13.5 ± 8.4	137.3 ± 27.1	98.4 ± 64.8	54.5 ± 14.1		62.7 ± 23.6
Male	59	13.8 ± 8.5	14.8 ± 8.8	135.7 ± 27.2	102.3 ± 64.3	53.3 ± 14.9		61.0 ± 23.1
Female	51	17.0 ± 8.2	11.9 ± 7.6	139.1 ± 27.3	93.8 ± 65.6	56.0 ± 13.0		64.6 ± 24.3

* Statistically significant compared to control group (*p* < 0.001)

** Statistically significant compared to control group (*p *< 0.03).

Total cholesterol, HDL‐C, and LDL‐C levels in all SCD patients were significantly (*p *< 0.001) lower than controls. Comparing SCD patients according to genotype with controls demonstrated that total cholesterol, HDL‐C, and LDL‐C were significantly lower in SS and S/β‐thalassemia patients compared to controls (*p *< 0.001) (Table 2).

There was a significant difference in total cholesterol, TG, and HDL‐C levels concerning the vitamin D levels. Table 3 indicates vitamin D levels were negatively correlated to TG (*p *= 0.039) and positively correlated to total cholesterol (*p *= 0.006) and HDL‐C (*p* < 0.001).

**TABLE 3:** Correlation of vitamin D level and lipid profile of patients and controls

Parameters mg/dl	Pearson Correlation	*p* Value	*R*‐square
Cholesterol	0.187**	0.006	0.035
Triglyceride	−0.141*	0.039	0.02
HDL‐C	0.254**	<0.001	0.065
LDL‐C	0.06	0.38	‐

** Correlation was significant at the 0.01 level

* Correlation was significant at the 0.05 level.

### 3.3 Genotyping

The distribution of FokI (SNPrs2228570) genotypes was in the Hardy‐Weinberg equilibrium in SCD patients (χ^2^ = 0.1, *p *> 0.1) and in controls (χ^2^ = 2.45, *p *> 0.1). Table 4 indicates the frequency of FokI genotypes and alleles in patients and controls. The frequencies of the C allele of FokI were 81.7% (*p *= 0.003), 80.2% (*p *= 0.034), and 84.6% (*p *= 0.011) in all SCD, SS, and S/β‐thalassemia patients, respectively, compared to 69.1% in controls.

**TABLE 4:** Distribution of FoKI (rs2228570) genotypes and alleles in SCD patients and controls

FokI genotypes	HbSS, *n* = 61 *n* (%)	S/β‐thalassemia, *n* = 39 *n* (%)	All patients, *n* = 101 *n* (%)	Controls, *n* = 110 *n* (%)
TT	2 (3)	1 (2.5)	3 (3)	7 (6.4)
TC	20 (33)	12 (30.8)	32 (32)	54 (49.1)
CC	39 (64)	26 (66.7)	66 (65)	49 (44.5)
	χ^2^ = 5.1^a^, *p *= 0.023,OR = 2.1 95% CI, (1.1–4.2, *p* = 0.024)	χ^2^ = 4.8^a^, *p* = 0.028 OR = 2.4, 95% CI, (1.1–5.2, *p *= 0.03)	χ^2^ = 8^a^, *p* = 0.005 OR = 2.3, 95% CI, (1.3–4.0, *p* = 0.005)	
Alleles				
T	24 (19.7)	14 (17.9)	38 (18.8)	68 (30.9)
C	98 (80.3)	64 (82.1)	164 (81.2)	152 (69.1)
	χ^2^ = 4.47, *p *= 0.034 OR = 1.75, 95% CI (1.03–2.96, *p *= 0.036)	χ^2^ = 6.5, *p *= 0.011 OR = 2.4, 95% CI (1.2–4.7, *p *= 0.012)	χ^2^ = 8.8, *p *= 0.003 OR = 2.0, 95% CI (1.3–3.1, *p *= 0.003)	

Note

Overall χ^2^ comparing three genotypes between all SCD patients and controls was 9.37, *p* = 0.009.

Overall χ^2^ comparing two alleles between all SCD patients and controls was 8.8, *p *= 0.003.

Overall χ^2^ comparing three genotypes between S/βthal patients and controls was 5.7, *p *= 0.056.

^a^ Compared to TC genotype between patients with HbSS and controls.

The frequencies of TaqI (rs731236) genotypes were in the Hardy‐Weinberg equilibrium in SCD patients (χ^2^ = 0.78, *p *> 0.1) and controls (χ^2^ = 0.61, *p* > 0.1). The distribution of TaqI genotypes and alleles among SCD patients and controls is depicted in Table 5. The frequencies of the C allele were 39.3% and 47.4% in SS and S/β‐thalassemia patients, respectively, compared to 36.8% in controls (*p* > 0.05).

**TABLE 5:** Distribution of TaqI (rs731236) and GC (rs7140) genotypes and alleles in patients and controls

TaqI genotypes	HbSS, *n* = 61 *n* (%)	S/β‐thalassemia, *n* = 39 *n* (%)	All patients, *n* = 101 *n* (%)	Controls, *n* = 110 *n* (%)
TT	23 (37.7)	9 (23)	32 (31.7)	42 (38.2)
TC	28 (46)	25 (64)	54 (53.5)	55 (50)
CC	10 (16.3)	5 (13)	15 (14.8)	13 (11.8)
Alleles				
T	74 (60.7)	41 (52.6)	118 (58.4)	139 (63.2)
C	48 (39.3)	37 (47.4)	84 (41.6)	81(36.8)

GC genotypes				
TT	9 (14.8)	12 (30.8)	21 (21)	25 (22.7)
TG	37 (60.7)	15(38.4)	52 (51)	53 (48.2)
GG	15 (24.5)	12 (30.8)	28 (28)	32 (29.1)
Alleles				
T	56 (45.9)	37 (47.4)	93 (46)	103 (46.8)
G	66 (54.1)	41 (52.6)	109 (54)	117(53.2)

Note

TaqI: Overall χ^2^ comparing three genotypes between all SCD patients and controls was 1.1, *p *= 0.57, Overall χ^2^ comparing two alleles between all SCD patients and controls was 1.0, *p *= 0.29.

GC: Overall χ^2^ comparing three genotypes between all SCD patients and controls was 0.24, *p *= 0.88, Overall χ^2^ comparing two alleles between all SCD patients and controls was 0.01, *p *= 0.91.

The distribution of GC (rs7041) genotypes was in the Hardy‐Weinberg equilibrium only in SCD patients (χ^2^ = 0.41, *p *> 0.1). The frequencies of the G allele were 54.1%, and 52.6% in SS and S/β‐thalassemia patients, respectively, and was 53.2% in controls (*p *> 0.05) (Table 5).

## 4 DISCUSSION

In the present study, around 94% of patients with HbSS and about 81% of S/β‐thalassemia patients had VDD. However, around 83% of healthy individuals had VDD. Our study demonstrated around 78% of SCA patients from Kurdistan of Iraq had severe VDD. The incidence of VDD is between 20% and 80% in some Middle Eastern counties. Lower vitamin D levels have been associated with lower hemoglobin and hematocrit levels and with higher reticulocyte counts among Egyptian children with SCD, suggested VDD might increase hemolysis of RBCs in these patients.^13^ In a study of 139 children (aged 7.9–15.1 years) with SCA, severe VDD (<10 ng/ml) was detected in 64.0% and only 2.2% had a sufficient level of vitamin D (>30 ng/ml). Moreover, vitamin D levels were associated with the pulmonary function.^14^ In patients with SCD, the prevalence of VDD is increased, which might exacerbate by enhanced erythropoiesis and basal metabolic rate, inadequate dietary intake, and reduced nutrient absorption due to inflammatory damage of the intestinal mucosa.^15^ It has been suggested that hemoglobin released by chronic hemolysis could contribute to vitamin D deficiency in SCD patients.^16^

Vitamin D should be carefully evaluated in SCA children because developing VDD in these children is higher than healthy individuals due to high melanin levels in the skin, low levels of physical activity, and low food intake in these children.^17^ Increased respiratory infections, muscle weakness, and enhance the risk of falls and microlesions are associated with VDD. Vitamin D deficiency was associated with bone weakness and painful crises and increased vitamin D level through supplementation was positively associated with functional and physical capacity.^17^ In a randomized clinical trial in pediatric patients with SCD, the potential benefit of vitamin D for preventing respiratory complications has been indicated with both monthly high‐ and standard‐dose vitamin D.^18^ The immunomodulatory function of vitamin D is through enhanced the innate immune response antimicrobial activity and reducing the adaptive immune response proinflammatory action^19^

Our study showed that plasma levels of total cholesterol, HDL‐C, and LDL‐C in SCD patients were significantly lower (*p *< 0.001) than controls and SCD patients had hypocholesterolemia. Hemolytic stress is associated with a significant reduction in total cholesterol, LDL‐C, and HDL‐C in SCA and sickle/β‐thalassemia patients compared to sickle cell trait and healthy individuals.^1^ A cohort study from the USA, studying 365 SCD patients and controls, demonstrated significantly decreased plasma levels of total cholesterol, HDL‐C, and LDL‐C in SCD patients compared to healthy controls.^20^ Also, previously, we reported lower total cholesterol, HDL‐C, and LDL‐C in SCD patients from Southern Iran compared to controls.^1^

In the present study, vitamin D levels were negatively correlated to TG and positively correlated to total cholesterol and HDL‐C. The levels of vitamin D have been negatively associated with TG and positively correlated to serum HDL‐C. In pediatrics patients with HbSS and S/β‐thalassemia and with low vitamin D levels significantly higher VLDL levels were detected compared to controls.^21^ Among SCD patients from Turkey, vitamin D levels were negatively correlated to TG and positively correlated to total cholesterol and HDL‐C.^21^ Inadequate vitamin D level is associated with chronic inflammation and also with low levels of HDL‐C as SCD patients with lower levels of vitamin D had lower HDL‐C values (<40 mg/dl). Low HDL‐C values could be considered as a prognostic marker of hemolysis and endothelial dysfunction.^4^ Also, irrespective of vitamin D status, SCD patients had significantly higher TG levels than controls, and hypertriglyceridemia in these patients was linked to chronic inflammation.^21^

In the present study, a significantly higher frequency of the FokI C allele was detected in patients with HbSS and S/β‐thalassemia patients than controls. In our study, the frequencies of the FokI CC genotype were around 64% and 66% in HbSS and S/β‐thalassemia patients, respectively. The frequency of the CC genotype of FokI was around 33% in Egyptian SCD children.^22^ In SCD children, the FokI polymorphism was associated with low bone mineral density at the forearm and lumbar spine and was a useful genetic marker in determining the bone mineral density and osteoporosis risk.^22^ Also, in β‐thalassemia major patients the polymorphism of FokI (FF or CC genotype) was significantly associated with the low bone mineral density of the lumbar spine and suggested that the VDR polymorphism can be used as an additional test in individuals susceptible to osteoporosis for early prevention.^23^ Since 1, 25 (OH) 2‐D3 through binding to VDR exerts its effect, the presence of polymorphism in the VDR gene might modulate the VDR expression. The C allele of VDR FokI polymorphism does not have the first ATG, and translation starts at the second ATG. So, the presence of this allele producing a shorter VDR protein by 3 amino acids compared with the T allele.^12^ The high prevalence of the FokI C allele along with the high prevalence of severe deficiency of vitamin D among our SCA patients should be considered as the serious risk factors for reduced bone mineral density and osteoporosis that ask for urgent supplementation of cholecalciferol in SCD patients from Kurdistan of Iraq. Vitamin D supplementation has improved the pain symptoms in an SCD patient with VDD and severe osteoporosis.^6^ In monozygotic twins (male or female pairs), supplementation of cholecalciferol at the concentration of 2000 IU for 2 months increased circulating serum vitamin D levels and VDR mRNA expression.^24^ Also, the VDR expression has been reported to be correlated with higher 25(OH)‐D levels suggested that the VDR was positively regulated by 1, 25‐(OH).^25^

In the present study, the frequencies of TaqI (rs731236) genotypes and alleles were not significantly different comparing SCD patients with controls. The absence of association between TaqI polymorphism with bone mineral density has been reported.^23^

In our study, we did not detect a statistically significant difference in the frequencies of genotypes and alleles of GC (rs7041) between SCD patients and controls. Also, an association between 25 (OH)‐D level and GC polymorphism was not observed in this study. Vitamin D binding protein that encoded by the GC gene binds to 25 (OH)‐D in plasma and serves as a reservoir and prolongs the 25 (OH)‐D half‐life.^25^ It has been reported that the GC (rs7041) polymorphism is related to a different binding affinity for 25 (OH)‐D. Reduced binding of 25 (OH)‐D to GC protein might decrease the levels of 25 (OH)‐D and other vitamin D metabolites. In some but not all studies, the GC polymorphism was significantly associated with lower serum levels of 25 (OH)‐D. The association of GC polymorphism with 25 (OH)‐D is ethnic dependent as it has been reported in some specific ethnic populations such as Arab and South Asian populations.^8^ In SCD patients, metabolic demands on the liver might reduce the capacity of vitamin D binding protein synthesis.^7^ Lower levels of 25 (OH)‐D levels were associated with decreased expression of GC and VDR genes.^25^

In conclusion, our study indicated the presence of hypocholesterolemia in patients with HbSS and S/β‐thalassemia from Kurdistan of Iraq. Also, we demonstrated a high prevalence of severe VDD in SCD patients from this area. Moreover, a significantly high frequency of the VDR FokI C allele was found among SCA and S/β‐thalassemia patients. Our study confirms induced hypocholesterolemia by hemolytic stress and positive association between vitamin D and HDL‐C levels that show adverse effects of low HDL‐C values on hemolysis in the presence of low vitamin D levels. On the other hand, VDD is associated with comorbidities and considering the high prevalence of VDR FokI C allele with an adverse effect on bone mineral density there is concern about children and adults with SCD patients that asks urgent and immediate intervention and supplementation of vitamin D in their diet.

### 4.1 Limitation of the study

The limitations of the present study were the low sample size of patients with S/β‐thalassemia and SD and the absence of studying sickle cell trait individuals.

## CONFLICTS OF INTEREST

The authors declare that they have no conflicts of interest.

## Data Availability

The data that support the findings of this study are available from the corresponding author upon reasonable request.
